# Why It’s Time to Reawaken Our Debates on the Aviation Analogy

**DOI:** 10.5334/pme.1399

**Published:** 2024-06-04

**Authors:** Sayra M. Cristancho

**Affiliations:** 1 Dept. of Surgery, Dept. of Anatomy and Cell Biology and Centre for Education Research & Innovation (CERI), The University of Western Ontario, Canada

It’s been a year since I had the hunch that I needed new glasses. Things had been getting blurry, but I refused to have my eyes checked. *“It’s clear enough”* was the story I kept telling myself. Looking back, that hesitancy reflects my human tendency to resist change. I loved my current glasses, and I hated the idea of having to adjust to a new pair. But then I fell while running: that blurry thing I thought was a tree leaf was in fact a rock. That fall ruined my months of biathlon training, so I had enough with blurriness. I went to the optometrist. And if you have ever done one of these appointments, you know how it goes: they get you down to the blurriest you can handle by changing the set of lenses, and then they bring you back to a level of crisp clarity. I am not fond of my new glasses (yet), but I love the clarity they afford me while running.

What does getting new glasses have to do with healthcare? It has to do with the analogies we have become accustomed to as we think about healthcare work. Those analogies can get blurry and may need reassessment and change to refocus us. Case in point, the aviation analogy.

In healthcare we’ve gotten very comfortable with the aviation analogy for thinking about teamwork and patient safety: we’ve heard it at conference podiums and read it in countless publications under the label of “the checklist manifesto” [1]. However, the more complex healthcare has become, the blurrier the aviation analogy has gotten. This blurriness has existed for a while; therefore, I am not suggesting this is a new claim. What I see is that depending on the societal moment, the debates about its *blurriness* have gotten louder or quieter. I argue that we are at a moment when these debates need to get louder again. Disruptions such as violence, public health, and climate events, are imposing new and unexpected challenges on healthcare systems that are demanding creative thinking. And to foster creative thinking we must rekindle the debates. That’s the gist of this piece.

We all might have different associations with the aviation analogy. For those involved in simulation, the analogy helps them promote leadership, for instance. The focus of my argument here, though, is around our uptake of the aviation analogy in relation to standardization, safety, and teamwork [2,3,4]. This uptake has prompted the creation of protocols and checklists for almost everything we do, from clinical practice (e.g., surgical safety checklist) to education (e.g., O-SCORE checklist). There’s nothing wrong with this: in fact, without the aviation analogy, we wouldn’t have made such robust progress in creating processes to help us learn from mistakes. However, the limits of its application to healthcare are important to recognize. For instance, healthcare professionals (HCPs) consult with patients before deciding on a course of action; air crews do not – their decisions on how to conduct a flight are not swayed by passengers’ expectations [5]. HCPs juggle safety and resilience; air crews do not – their only priority is safety [6]. HCPs bypass protocols to serve a patient’s best interests, even if they must consider less than ideal outcomes; air crews do not – their only acceptable outcome is to deliver an uneventful journey [4]. HCPs perform tasks and roles that overlap considerably; air crews do not – pilots and flight attendants have markedly distinct role responsibilities [3].

The aviation analogy became particularly blurry during the Covid-19 pandemic. Despite the pervasive use of checklists, akin to aviation, many asked why very few HCPs seemed capable of dealing with the disruption caused by Covid? What made those few better at it? Was it their heavy reliance on their checklists? Or was it the opposite: their ability to step away from checklists? In an interview study we explored those questions with front-line physicians and nurses [7]. They all confirmed the latter, either in themselves or in others who led them. We learned that their need to step away from checklists came from a sense that they were not required to adapt to *make things better*. They were required to adapt to *survive*. For many, it was not a successful adaptation, it was an unexpected implosion. As front-line physicians and nurses remarked, checklists become impractical when situations implode and priorities conflict. ‘How can we aspire to be both safe and resilient?’ was a question they constantly asked among themselves. That’s the point when the aviation analogy begins to blur our vision instead of sharpening it.

As I started to tease out this process of implosive adaptation, I wondered if the problem with analogies is about the mindset they promote. For instance, checklists can promote a mindset of role separation: I am a physician, you are a nurse; as long as you do your job and I do mine, we will be fine [8]. During the pandemic, that mindset collapsed when people started to be redeployed. The idea of HCPs doing tasks outside their role or outside their usual care setting was paralyzing to many, which affected teamwork and morale and diminished the resilience of the whole system. Even though some embraced redeployment, they were not the majority. Those who did seemed to have a different mindset. In our exploration, HCPs who had worked in rural or small hospitals, for instance, seemed more comfortable with, rather than surprised, by unexpected disruption. They saw it frequently and therefore they thought about it constantly.

If we are to move beyond the powerful and entrenched aviation analogy, what could possibly be credible to take its place? I wondered if we could find other contexts where implosive adaptation is the norm, not the exception, so that we might be able to borrow some insights. And I found it in the resilience of nature, particularly social insects. To respect the conciseness of the commentary genre, I will provide a brief description of the analogy in the sentences that follow and invite readers to consider the in-depth illustration I recently offered in PME [9].

For social insects, unexpected disruption is, well, to be expected. And collective response is the only way to survive. When attacked, a single bee can only release alarm pheromones to ask for help, but a beehive can deliver a precise counterattack [10]. The mindset of embracing a collective response to unexpected disruption that HCPs in those rural and small hospitals were describing, made me think of a beehive or an ant colony. That’s when a new analogy felt like a fresh, new pair of glasses. Through this new pair of glasses, role separation was still in sight. Bees and ants have a clear division of labour that they abide by during times of stability. However, for bees and ants, role separation is plastic, not rigid. When unexpected disruption happens, role separation disappears as bees and ants collectively reorganize by taking on other bees’ and ants’ tasks as needed [11]. Furthermore, when unexpected disruption happens, bees and ants don’t hesitate to move their colonies to a different place – i.e., to go where their work is required for conserving the environment. It appears as though the behaviour of social insects might provide an instructive example of how to balance safety and resilience [6].

These ideas are not something that the aviation analogy is particularly effective at instilling. Rather a biological analogy might help. When a disruption threatens survival, bees and ants don’t hesitate to act right away because they are programmed – hence my use of the word “mindset” – to be plastic. In other words, they accept that the goal of maintaining *existence* of function (a.k.a. ecological resilience) takes over the goal of maintaining *efficiency* of function (a.k.a engineering resilience) when the unexpected happens [12].

If we are to maintain existence of healthcare function despite disruptions, here I suggest that moving forward we need to question the comforts of the aviation analogy. I offer the following three comforts to spark conversation:

- The comfort of standardization: In aviation, standardization, through the use of checklists, is unswervingly embraced by air crews to streamline the complexities of flight. In healthcare, the use of checklists to promote standardization has evolved into a contentious matter, burdening healthcare teams with added workload [2]. When disruptive events strike, our fervent insistence on exhaustive checklists can undermine the advantages of agile improvisation, potentially placing our crisis response at risk.
- The comfort of safety: In aviation, safety is an all-encompassing priority, woven into every level of operation, as both air crew and passengers equally suffer the consequences of any adverse outcome. In healthcare, safety no longer reigns supreme [13]; it must be delicately negotiated when resilience is the imperative [6]. During disruptive events, an unrelenting pursuit of safety can prove paralyzing, especially when confronted with the need to embrace some uncomfortable level of risk.
- The comfort of teamwork: In aviation, teamwork looks different depending on who’s on the team, but not on who the passengers are or where they are seated. In healthcare, teamwork isn’t solely contingent on the composition of the team, it is intrinsically tied to the patient’s identity and their location. During disruptive events, such as mass casualties, traditional models of teamwork are upended: non-medically trained individuals might become part of the team, teams might need to be deployed to non-hospital locations, and team members might find themselves doing tasks outside their scope. Working despite teamwork instability then becomes the required approach.

Creative analogies are useful to inspire new insights about issues. The aviation analogy has offered tremendous intellectual nourishment in our attempts at making healthcare safer and more reliable. However, the demands and complexity of healthcare since the early 2000s have exposed the misalignment of those two aims [14]. This misalignment, therefore, has resulted in debates that question the aviation analogy [5,15]. These debates aren’t new. But the challenges healthcare systems now face are. Not only are healthcare systems required to cope with ageing populations alongside massive technological and economic forces; they now must also respond to wider societal crises such as pandemics, terrorism, and climate disasters [16]. So, if the latter challenges are now part of the forces that are “causing the tectonic plates of healthcare to shift in ways not yet fully appreciated” [17,18], why do we still cling to the aviation analogy?

Wider societal crises are forcing us to think differently about the relationship between safety and resilience. To think differently, we must question established analogies and embrace new ones. And in doing so, I am now wondering if the time has come to abandon some of the comfort of the aviation analogy and seek new and complementary analogies that will be both uncomfortable and, hopefully, productive. In doing that, we might have a chance at revitalizing healthcare in these times of change and disruption.
